# Hypoxia and hyperglycaemia determine why some endometrial tumours fail to respond to metformin

**DOI:** 10.1038/s41416-019-0627-y

**Published:** 2019-12-10

**Authors:** Vanitha N. Sivalingam, Ayşe Latif, Sarah Kitson, Rhona McVey, Katherine G. Finegan, Kay Marshall, Michael P. Lisanti, Federica Sotgia, Ian J. Stratford, Emma J. Crosbie

**Affiliations:** 1 Division of Cancer Sciences, School of Medical Sciences, Faculty of Biology, Medicine and Health, University of Manchester, St Mary’s Hospital, Manchester, UK; 2 Department of Obstetrics and Gynaecology, Manchester University Hospitals NHS Foundation Trust, Manchester Academic Health Science Centre, Manchester, UK; 3 Division of Pharmacy and Optometry, School of Health Sciences, Faculty of Biology, Medicine and Health, University of Manchester, Manchester, UK; 4 Department of Histopathology, Manchester University Hospitals NHS Foundation Trust, Manchester Academic Health Science Centre, Manchester, UK; 5 School of Environmental & Life Sciences, University of Salford, Salford, UK

**Keywords:** Endometrial cancer, Cancer metabolism

## Abstract

**Background:**

High expression of Ki67, a proliferation marker, is associated with reduced endometrial cancer-specific survival. Pre-surgical metformin reduces tumour Ki-67 expression in some women with endometrial cancer. Metformin’s anti-cancer activity may relate to effects on cellular energy metabolism. Since tumour hypoxia and glucose availability are major cellular redox determinants, we evaluated their role in endometrial cancer response to metformin.

**Methods:**

Endometrial cancer biopsies from women treated with pre-surgical metformin were tested for the hypoxia markers, HIF-1α and CA-9. Endometrial cancer cell lines were treated with metformin in variable glucose concentrations in normoxia or hypoxia and cell viability, mitochondrial biogenesis, function and energy metabolism were assessed.

**Results:**

In women treated with metformin (*n* = 28), Ki-67 response was lower in hypoxic tumours. Metformin showed minimal cytostatic effects towards Ishikawa and HEC1A cells in conventional medium (25 mM glucose). In low glucose (5.5 mM), a dose-dependent cytostatic effect was observed in normoxia but attenuated in hypoxia. Tumours treated with metformin showed increased mitochondrial mass (*n* = 25), while in cultured cells metformin decreased mitochondrial function. Metformin targets mitochondrial respiration, however, in hypoxic, high glucose conditions, there was a switch to glycolytic metabolism and decreased metformin response.

**Conclusions:**

Understanding the metabolic adaptations of endometrial tumours may identify patients likely to derive clinical benefit from metformin.

## Background

Endometrial cancer is the most common gynaecological malignancy in developed countries. In the United Kingdom, the incidence has almost doubled since the 1990s,^1^ driven mainly by the obesity epidemic.^2^ Similar trends have been reported in the United States, with an estimated 60,500 new cases in 2016.^3^ Insulin resistance often co-exists with obesity and is an independent risk factor for endometrial cancer. Surgical treatment of early stage endometrial cancer is usually curative, but is accompanied by increased peri-operative morbidity in obese women.^4^ Current non-surgical alternatives offer lower rates of cure and are associated with a high risk of relapse.^5,6^ Thus, there is a need to explore new, efficacious, non-surgical approaches for those unsuitable for surgery, those who wish to avoid a hysterectomy for fertility reasons, as well as for women with advanced, recurrent endometrial cancer.

Metformin, long used in the treatment of type 2 diabetes, has shown promise as an anti-cancer drug in endometrial cancer.^7^ We found that short-term pre-surgical treatment with metformin reduced tumour proliferation in some non-diabetic women with endometrioid endometrial cancer. The response was measured by change in Ki-67, a cellular proliferation marker, and individual women varied in their response.^8^ In exploratory subgroup analyses, associations with body mass index were observed; leaner women showed a greater Ki-67 change post-treatment than obese women.^8,9^ Heterogeneity of response to metformin has been documented in other pre-surgical window studies in endometrial,^9–12^ breast^13–15^ and prostate cancer^16^ and is likely to be related to both patient- and tumour-associated factors.

In type 2 diabetes, metformin increases insulin sensitivity and reduces serum glucose concentration through enhanced myocyte-driven glucose uptake and inhibition of hepatic gluconeogenesis.^17^ At a cellular level, metformin is a mitochondrial toxin that affects energy balance through inhibition of mitochondrial electron transport, leading to a reduction in oxidative phosphorylation.^18^ Higher doses of metformin or combination therapy are required for obese patients with severe type 2 diabetes and extreme hyperglycaemia.^19^ A hallmark of cancer is metabolic reprogramming with an increased consumption of glucose and glutamine.^20^ Cancer cells, however, can alter their metabolism to adapt to decreased oxygen availability in the microenvironment with the activation of certain genes including hypoxia-inducible factor-1α (HIF-1α) and its downstream target, carbonic anhydrase 9 (CA-9).^21^ Thus, hyperglycaemia and local hypoxia may drive resistance to metformin. One such resistance mechanism is a preferential switch from oxidative phosphorylation to glycolytic metabolism.^22^ We, therefore, hypothesised that the anti-cancer activity of metformin could vary according to tissue glucose and oxygen availability. The objective of our work was to establish metabolic determinants of metformin response, with the ultimate aim of identifying endometrial cancer patients most likely to derive clinical benefit from metformin.

## Methods

### Cell lines, cell culture and treatments

Ishikawa (99040201, HPA Culture Collections, Salisbury, UK) and HEC1A (HTB-112, ATCC, Middlesex, UK) cells were maintained in DMEM growth medium (LTC, Paisley, UK) supplemented with 10% heat-inactivated FBS and Glutamax. Ishikawa and HEC1A cells were originally established from well differentiated^23^ and moderately differentiated^24^ endometrioid endometrial adenocarcinomas, respectively. Cells used in experiments were at passage 5–8 (post purchase of frozen stocks). For cells used beyond this early passage, they were authenticated during and at the end of the study using the DNA sequencing facility the Cultures Collection, Public Health England. Cells were tested every 3–4 weeks for mycoplasma and shown to be negative throughout the study. For in vitro studies, metformin (1,1-dimethylbiguanide hydrochloride) (Sigma-Aldrich, Dorset, UK) was diluted in a PBS stock solution and used at concentrations detailed.

### Cytostatic assays

For cytostatic assays, Ishikawa and HEC1A cells were plated in 96-well plates at concentrations of 5000 and 10,000 cells/well, respectively. Cells were treated in DMEM with low (5.5 mM) or high (25 mM) glucose, subsequently referred to as low and high glucose. These two concentrations of glucose were chosen because standard growth media contains 25 mM glucose and most previous studies of the action of metformin in vitro have been carried out in media containing this glucose concentration.^25–28^ However, 25 mM glucose in vitro is comparable with extreme hyperglycaemia in humans, while 5.5 mM glucose in vitro is more consistent with normoglycaemic serum concentrations. For hypoxic conditions, cells were incubated in hypoxia (1% O_2_) for 24 h and then treated for a further 24–72 h in hypoxia with metformin-supplemented media or vehicle alone. After treatment, cells were fixed with 10% (w/v) trichloroacetic acid and incubated with sulforhodamine B (SRB) for 15 min, followed by 1% (v/v) acetic acid washing and drying steps. Finally, the protein-bound dye was dissolved in 10 mM Tris at pH 8.8 and optical density read at 540 nm.^29^ The optical density is directly proportional to cell number and results are presented as a percentage of cells in treated groups compared with untreated controls.

### Mitochondrial flow cytometric assay

Fluorescent-activated cell sorting (FACS) was used to assess mitochondrial mass and function in Ishikawa and HEC1A cells plated in 6 well plates at 20,000 and 30,000 cells/well, respectively. Cells were cultured in low and high glucose media for 24 h, then treated with 2 mM metformin (chosen as it showed less than 50% cytostatic behaviour in both cell lines in high and low glucose) or vehicle alone for 72 h.

MitoTracker Deep Red (M22426) (Molecular Probes, UK) and Mitotracker Orange CMTMRos (M7510) (Molecular Probes, UK) were used to measure mitochondrial mass and function in live cells. A change in the mitochondrial membrane potential, as assessed by MitoTracker Orange CMTMRos was used as a measure of change in mitochondrial function. Cells were incubated in MitoTracker staining solution [50 nM MitoTracker in PBS (phosphate-buffered saline0 with added MgCl_2_/CaCl_2_], prior to washing with PBS, harvesting and sorting for flow cytometric analysis (Fortessa, BD Bioscience, UK). Flowjo software was used to identify dead cells, couplets and debris, ensuring that cells analysed were single cells.

### Extracellular flux analysis

Cellular mitochondrial function was additionally measured using a Seahorse XF^e^96 Analyser and a XF Cell Mito Stress Test (Agilent Technologies, Santa Clara, USA). Ishikawa and HEC1A cells were incubated in low and high glucose media, supplemented with L-glutamine. Cells in 96 well plates were treated for 24 h with 2 mM metformin or vehicle alone in low and high glucose media, concurrently in normoxia or hypoxia (3% O_2_). An oxygen tension of 3% is the lowest at which the extracellular flux analysis provides reproducible measurements of oxygen consumption. Cell numbers were optimised to provide reproducible changes in oxygen consumption rate (OCR) and restoration to the ambient oxygen concentrations between measurements (normoxia or 3% O_2_; Supplementary Figs. 1 and 2). For hypoxia assays, all manipulations including the Seahorse analyses were carried out under hypoxic conditions using the Whitley H35 Hypoxystation and Whitley i2 Instrument Workstation (Don Whitley Scientific, Bingley, UK). A mixing step was used to restore oxygen levels between each measurement cycle to ensure reliable repeated measurements of OCR in normoxia and hypoxia (3% O_2_). Maintenance of hypoxia was confirmed using a NaSO_3_ control. (Supplementary Fig. 2).

During the XF Cell Mito Stress Test, OCR (pMoles O_2_ consumed/minute) and ECAR (extracellular acidification rate, measured as change in mpH units/min) could be measured simultaneously. After replacement of growth media with Seahorse buffer containing metformin or vehicle, the mitochondrial function was assayed by sequential injections of oligomycin (ATP synthase inhibitor), FCCP (carbonylcyanide p-trifluoromethoxy phenylhydrazone), mitochondrial oxidative phosphorylation protonophore and uncoupler, rotenone (mitochondrial complex I inhibitor) and antimycin A (mitochondrial complex III inhibitor) (Agilent Technologies, Santa Clara, US). This allowed us to define basal OCR, ATP-linked OCR, proton leak, maximal respiratory capacity, reserve respiratory capacity and non-mitochondrial oxygen consumption. To normalise the results, cell number/well was quantified using the SRB assay at the end of the recording.

After normalising to cell numbers, ECAR and OCR values were and plotted as mean ± SEM. A schematic representation is given in Supplementary Fig. 1. Basal ECAR (basal glycolytic and non-glycolytic acidification) was measured during the assessment of basal respiration in cells already conditioned in glucose to determine the effect of metformin pre-treatment on glycolysis. ECAR measured following oligomycin, which effectively shuts down oxidative phosphorylation represents maximum glycolytic capacity, ECAR measured after rotenone and antimycin A injections represent non-glycolytic acidification. This allows calculation of glycolytic reserve; i.e. the capacity of a cell to respond to energetic demand (Supplementary Fig. 1).

Glycolytic reserve = maximum ECAR (glycolytic capacity + non-glycolytic acidification) − basal ECAR (glycolysis + on glycolytic acidification)

### RNA extraction and real-time PCR

The expression of hypoxia-dependent genes (VEGF, GLUT-1 and CA-9) was quantified using qRT-PCR to confirm hypoxia induction at 3% O_2_ levels. Total RNA was extracted from fixed cells following extracellular flux analysis according to the PrimeScript TMRT reagent kit (Ambion RNA, Thermo-Fisher Scientific, UK). Extracted RNA was subjected to a DNA-free reaction and converted to cDNA via reverse-transcription. Subsequent cDNA was analysed by real-time PCR using the Applied Biosystems 7000 Sequence Detection System. Detailed methodology is available in the supplementary information. All real-time experiments were carried out in duplicate and at the end of the PCR the calculated cycle threshold (CT) values were analysed. Relative mRNA levels of vascular endothelial growth factor (VEGF), Carbonic Anhydrase-9 (CA-9) and Glucose Transporter-1 (GLUT-1) from the two different cell lines were normalised to levels of hypoxanthine-guanine phosphoribosyltransferase (HPRT), which does not change following hypoxic exposure.

### Pre-surgical window study

Approval was received from the North West Centre for Research Ethics Committee and all participants provided informed consent. Women with atypical endometrial hyperplasia or endometrioid endometrial adenocarcinoma received metformin 850 mg twice daily (*n* = 28) or no drug (*n* = 12) in the pre-surgical window (median 20 days) between diagnosis and hysterectomy. Endometrial cancer tissue proliferation markers, serum markers of insulin resistance and anthropometrics were measured before and after the pre-surgical window as previously described.^8^

### Immunohistochemistry

Hypoxia-inducible factor-1α (HIF-1α) and CA-9 expression were assessed on tissue microarrays (TMAs) (*n* = 40). TMAs were created from triplicate cores taken from equivalent areas in the pre- and post-intervention biopsies by the study histopathologist who was blinded to treatment arm, as previously described.^8^ Selection was not based on vascular “hot spots”, and instead provided tumour representative of equivalent grade in the paired samples. Areas of tumour necrosis were avoided. Immunohistochemistry was performed on paraffin-embedded tissue sections using the Leica Bond Max (Leica Biosystems, Wetzlar, Germany). Ki-67 staining and scoring has been described previously.^30^ Sections were de-paraffinised, rehydrated and underwent heat-induced epitope retrieval unless stated. Full details of the antibodies and conditions are given in Supplementary Table 1. Primary antibodies used were HIF-1α (BD 610959, BD Biosciences, UK) and CA-9 (NB100-147, Novus Biological, Colorado, US). Translocase of the outer mitochondrial membrane 20 (TOMM20) (#sc-17764, Santa Cruz Biotech, Dallas, US) staining was performed on whole sections in women where sufficient pre- and post-intervention tissue was available (*n* = 33/40). TOMM20 expression has been shown to correlate with mitochondrial mass and function.^31^ The primary antibody detection was performed using a post-primary solution and polymer detection which utilised a rabbit anti-mouse IgG secondary and anti-rabbit poly HRP IgG antibodies. This was followed by 3,3’-diaminobenzidine (DAB) staining as a chromogen (Refine Detection Kit, Leica Biosystems, Wetzlar, Germany).

Slides (TMAs and whole sections) were digitised (Leica SCN400 Slide Scanner, Wetzlar, Germany) and all malignant glands within the triplicate cores were scored. HIF-1α (nuclear) CA-9 (cytoplasmic and membrane) and TOMM20 (cytoplasmic) staining were assessed using a modified H-score, calculated as the product of the area of positive staining (scored 0–6) and the intensity of staining (scored 0 = none, 1 = mild, 2 = moderate, 3 = strong), giving a maximum of 18. All scoring was performed by independently by two separate individuals who were blinded to the treatment group. The inter-observer intra-class correlation coefficient was 0.98 (95% CI 0.95-0.99), 0.92 (95% CI 0.77-0.96) and 0.91 (95% CI 0.67-0.96) for HIF-1α, CA-9 and TOMM20, respectively. Any discrepancies of greater than 10% were reviewed collectively and resolved by consensus agreement.

### Statistical analysis

All statistical analyses were performed using GraphPad Prism version 6.0 and IBM SPSS Statistics version 23.0. Cell culture experiments were carried out in triplicate on three independent occasions (unless otherwise stated). Data are presented as mean ± SEM. Parametric data were analysed using unpaired Student’s *t*-tests, one and two-way analysis of variance (ANOVA). The treatment effect on Ki-67 and IHC markers was tested using an analysis of covariance linear model with post-treatment score as the response variable, adjusting for covariates (baseline Ki-67, age, body mass index, tumour grade) and change in controls. Statistical significance was accepted at *p* < 0.05. In the figures, N/S, *, **, and ***, **** indicates not-significant, *p* < 0.05, 0.01, 0.001, and 0.0001, respectively.

## Results

### Tumour hypoxia affects metformin response in vitro and in vivo

**Fig. 1:**
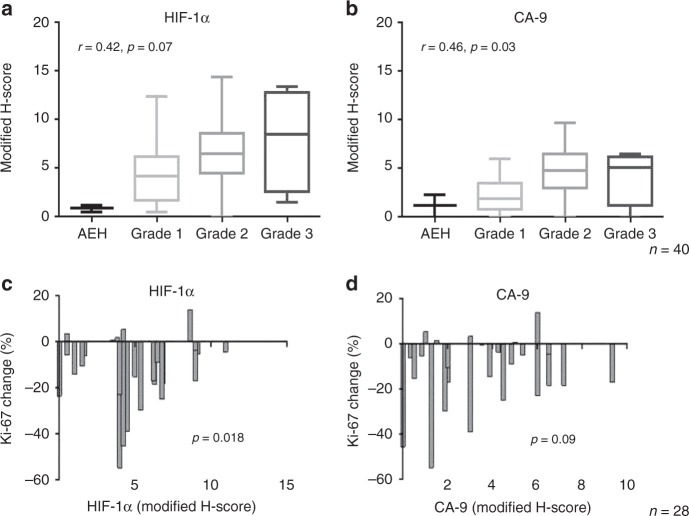
Tumour hypoxia as assessed by high expression of HIF-1α and CA-9 affects metformin response in vivo. Increased tumour hypoxia is positively correlated with grade using **a** HIF-1α (*r* = 0.42, *p* = 0.07) and **b** CA-9 (*r* = 0.46, *p* = 0.03) IHC expression (*n* = 40 women). Numbers of tumours in each grade are: 2 atypical hyperplasia (AEH), 15 grade 1, 19 grade 2 and 4 grade 3. Change in Ki-67 (i.e. response to metformin) was correlated with baseline **c** HIF-1α and **d** CA-9. The figures illustrate the change in Ki-67 in endometrial tumour biopsies taken pre- and post-metformin treatment in 28 patients. Response to metformin was lower in hypoxic tumours as assessed by HIF-1α [mean adjusted difference 2.5% (95% CI 0.4, 4.6%), *p* = 0.018] and approached statistical significance with CA-9 [mean adjusted difference 10.9% (95% CI -2.0, 23.7%, *p* = 0.09) using an analysis of covariance

Pre- and post-intervention tumour biopsies were obtained in the context of a pre-surgical window study of metformin in women with endometrial cancer.^8^ Baseline tumour hypoxia was assessed in pre-intervention biopsies (*n* = 40 women) and was comparable in metformin-treated (*n* = 28) and control (*n* = 12) patients. Tumour hypoxia, as measured by high expression of HIF-1α (*r* = 0.42, *p* = 0.07) and CA-9 (r = 0.46, *p* = 0.03), was positively correlated with tumour grade (atypical hyperplasia, *n* = 2, grade 1, *n* = 15, grade 2, *n* = 1 and grade 3, *n* = 4) (Fig. 1a, b) and both hypoxia markers positively correlated with each other (*r* = 0.45, *p* = 0.004). We previously showed that short-term oral metformin reduces cellular proliferation in some patients with endometrioid endometrial cancer, as measured by a reduction in post-intervention Ki-67 expression. The Ki-67 response was significantly lower in tumours with high HIF-1α expression [mean adjusted difference −2.5% (95% CI −0.4, -4.6%) *p* = 0.018], using an ANCOVA adjusting for baseline HIF-1α, baseline Ki-67, BMI and tumour grade (Fig. 1c, d). There was also a trend towards a lower Ki-67 response in tumours with high CA-9 (not statistically significant). Low grade tumours showed the greatest Ki-67 response to metformin, after adjusting for hypoxia [mean adjusted difference −10.4% (95% CI 0.7, 20.1%) *p* = 0.037].

**Fig. 2:**
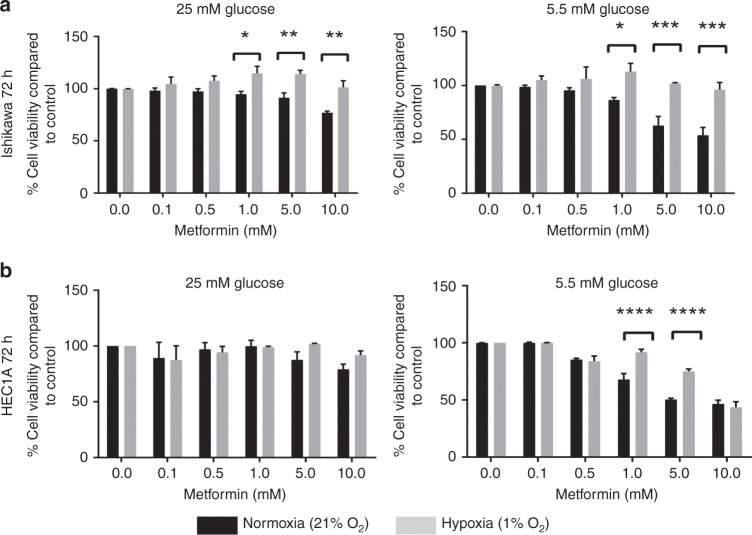
Hypoxia and high glucose reduce sensitivity of EC cells to metformin. **a** Ishikawa cells grown in low and high glucose were more sensitive to the cytostatic effects of metformin in normoxia (*n* = 3 biological replicates). **b** HEC1A cells grown in low glucose were more sensitive to the effects of metformin in normoxia. The bars represent the percentage cell viability ± SEM compared with untreated controls (*n* = 3 biological replicates)

In order to further demonstrate the role of hypoxia and hyperglycaemia in metformin response, two endometrioid endometrial cancer cell lines, Ishikawa and HEC1A cells were treated with metformin in the presence of low (5.5 mM) or high (25 mM) glucose concentrations in either normoxia (21% O_2_) or hypoxia (1% O_2_). Metformin treatment for 72 hours in low glucose in normoxia resulted in a dose-dependent inhibition of cell viability in both cell lines (Fig. 2). The cytostatic effects of metformin were less apparent in high glucose media and in hypoxia. This decrease in response was more pronounced in Ishikawa cells, in both high and low glucose (Fig. 2a, *p* < 0.05–0.001), while the protective effects of hypoxia were only noted in low glucose in HEC1A cells (Fig. 2b, *p* < 0.0001).

Short-term exposures to metformin (24 h) elicited no significant cytostatic effects, even in low (5.5 mM) glucose. However, when exposing Ishikawa cells to ultra-low glucose concentrations (0.5 and 1 mM) for 24 h, a metformin dose-dependent cytostatic effect was observed and these effects on cell viability were again attenuated in hypoxia (Supplementary Fig. 3).

### Metformin treatment reduces mitochondrial function with a compensatory increase in mitochondrial biogenesis

**Fig. 3:**
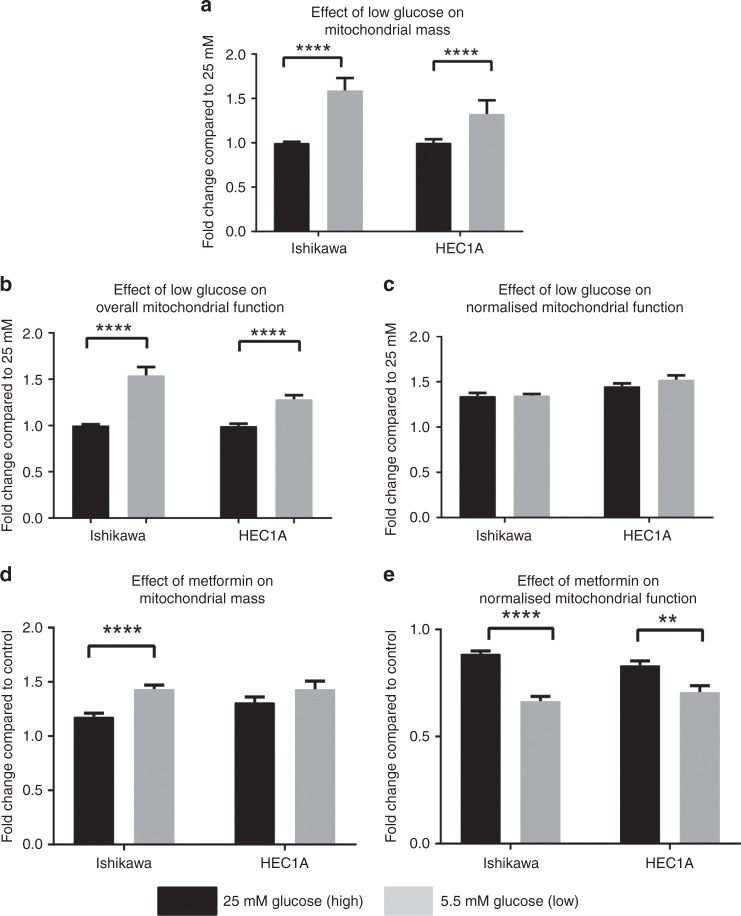
Upper panel: Endometrial cancer cell lines have an increased mitochondrial mass in low glucose. **a** Ishikawa and HEC1A cells grown in low glucose (grey bars) have increased mitochondrial mass compared with cells in high glucose (black bars). **b** Overall mitochondrial function was decreased in cells grown in high glucose, compared with low glucose, however, **c** after adjusting for increased mitochondrial mass, there was no change in mitochondrial function in Ishikawa and HEC1A cells. The bars represent the fold-change ± SEM compared with high glucose (*n* = 3 biological replicates). Lower panel: Metformin reduces endometrial cancer cell mitochondrial function in both high and low glucose. EC cells were treated in high (25 mM, black bars) and low (5.5 mM, grey bars) glucose media with or without 2 mM metformin for 72 h. The bars represent fold-change ± SEM compared with untreated cells in cultured in high and low glucose. In Ishikawa and HEC1A cells, mitochondrial mass was increased in both high and low glucose (**a**), while mitochondrial function was decreased (**b**) (*n* = 3 biological replicates)

We evaluated the effect of metformin on mitochondrial biogenesis and function in cells cultured in high and low glucose concentrations. First, the impact of varying glucose concentrations on mitochondrial biogenesis was assessed. Cells grown for 72 h in low glucose had an increased mitochondrial mass compared with cells grown in high glucose (Fig. 3a, *p* < 0.0001). There was no change in overall mitochondrial function after normalising for increased mitochondrial mass (Fig. 3c).

Cells were then treated with metformin in high and low glucose concentrations. Exposure to metformin resulted in an increase in mitochondrial mass in the endometrial cancer cells relative to untreated controls in both the high and low glucose-containing media (Fig. 3d, *p* < 0.0001). However, after adjusting for this increase in mitochondrial mass, there was a clear and significant decrease in mitochondrial function (as measured by a decrease in mitochondrial membrane potential) in both cell lines at both glucose concentrations (Fig. 3e, *p* < 0.01–0.0001). The decrease in function was greatest under the low glucose conditions. In the Ishikawa and HEC1A cells the metformin-induced reduction in mitochondrial function compared to controls was 34 and 29% in low glucose compared to reductions of 11 and 17% in high glucose, respectively.

**Fig. 4:**
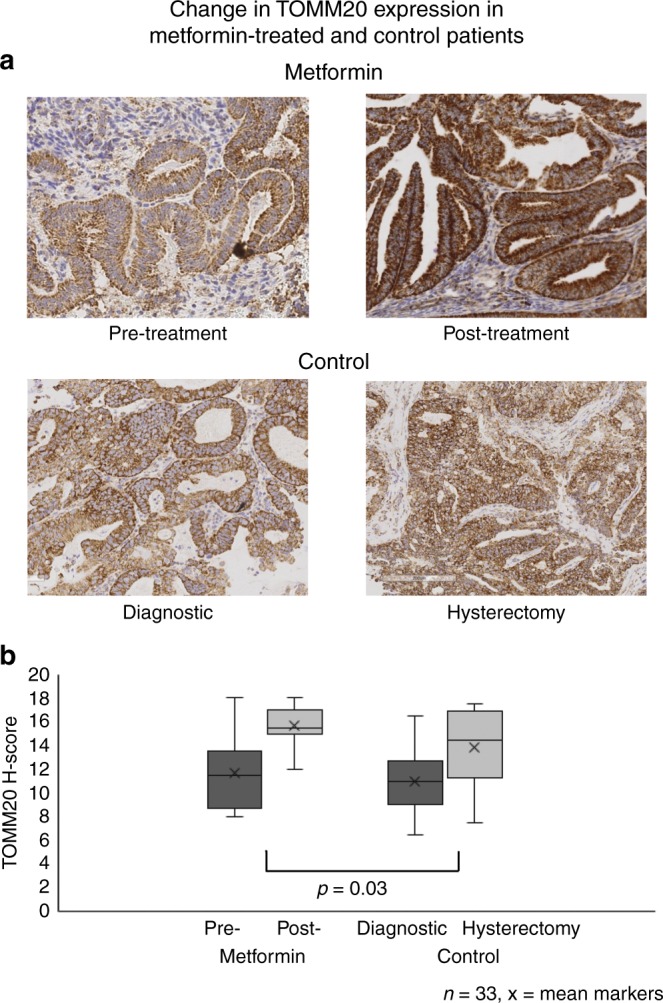
Metformin increases mitochondrial mass in endometrial tumours. **a** Endometrial tumours stained for TOMM-20 (translocase of the outer mitochondrial membrane), before and after metformin treatment (*n* = 25) and in the control group (at diagnosis and hysterectomy) (*n* = 8). Representative Images taken at ×10 magnification. **b** Box-and-whisker plots showing the change in TOMM-20 in metformin-treated and control patients. The ANCOVA showed an increase of 2.1 (95% CI 0.2–4.0, *p* = 0.03) in TOMM20 expression in metformin treated patients after adjusting for baseline expression, age, BMI, tumour grade and change in controls

While it is difficult to assess equivalent metformin-induced changes in mitochondrial function in vivo, we were able to evaluate mitochondrial mass. To do this we measured cytoplasmic TOMM20 expression in the endometrial cancer biopsies taken at recruitment to the pre-surgical window study compared with tissue harvested at hysterectomy. TOMM20 was noted to selectively stain the epithelial endometrial cancer cells and was largely excluded from the adjacent tumour stromal compartment (Fig. 4a), thus demonstrating a relative increase in mitochondria in epithelial tumour compared with stromal cells. Additionally, baseline TOMM20 expression showed a trend towards a positive correlation with increasing tumour grade [Spearman’s correlation *r* = 0.33 (95% CI −0.2, 0.63), *p* = 0.06]. The mean baseline scores of metformin treated (*n* = 25) and control tumours (*n* = 8) were similar, 11.6 ± 2.8 and 11.0 ± 3, respectively (Fig. 4b). However, TOMM20 expression increased by 2.1 (95% CI 0.2–4.0, *p* = 0.033) in the hysterectomy samples taken post metformin treatment compared with baseline. This was derived after adjusting for baseline expression, age, BMI, tumour grade and change in the control tumours. Thus, short-term metformin treatment is shown to directly increase mitochondrial mass in women with endometrioid endometrial cancer.

### Hypoxia and hyperglycaemia reduce the effect of metformin on mitochondrial oxidative phosphorylation

We determined changes in mitochondrial function following metformin treatment using the Seahorse metabolic analyser. Experiments were carried out in normoxia (21% O_2_) and hypoxia (3% O_2_) in the presence of high or low glucose concentrations. We confirmed that exposure of endometrial cancer cells to 3% O_2_ for 24 hours was sufficient to increase the expression of hypoxia-dependent genes (CA-9, VEGF and Glut-1) (Supplementary Fig. 4).

**Fig. 5:**
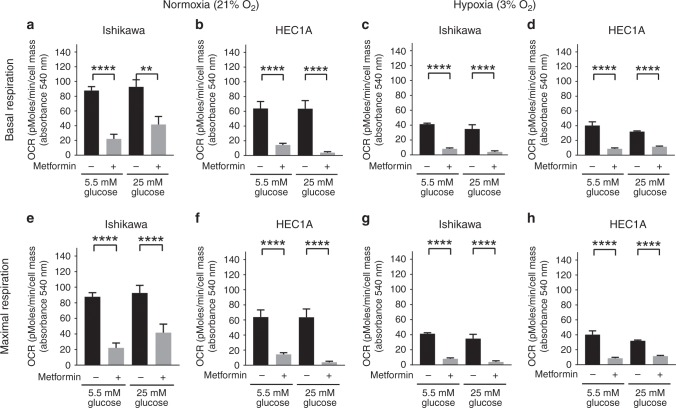
Hypoxia and hyperglycaemia reduce the effect of metformin on mitochondrial oxidative phosphorylation. Ishikawa and HEC1A cells were exposed to different concentrations of glucose (5.5 mM vs 25 mM) and oxygen (21% vs 3%) with or without 2 mM metformin for 72 h. Grey and black bars represented mean ± SEM with and without metformin treatment, respectively. In normoxia (21% O_2_), treatment with metformin impaired basal (**a**, **b**) and maximal respiration (**e**, **f**) in low or high glucose. In hypoxia (3% O_2_), a reduction in basal (**c**, **d**) and maximal (**g**, **h**) respiration was observed in both glucose concentrations (*n* = 3 biological replicates)

Ishikawa and HEC1A cells in media containing high or low glucose were treated for 72 h in normoxia or hypoxia in the presence or absence of 2 mM metformin. We observed similar basal respirations rates in both glucose concentrations in Ishikawa and HEC1A cell lines in normoxia. Treatment with metformin impaired basal respiration of Ishikawa cells by 75 and 65% in low and high glucose, respectively. In HEC1A cells, metformin caused an almost 100% drop in respiration at both glucose concentrations (Fig. 5a, b). In hypoxia (Fig. 5c, d), the cell lines showed reduced mitochondrial basal respiration in both glucose concentrations. Treatment with metformin further impaired oxidative phosphorylation for both cell lines. The changes in maximal respiration in normoxia and hypoxia, at low and high glucose and following treatment with and without metformin, generally parallel those changes seen in basal respiration (5 E-H). The accompanying data for effects on Proton leak and ATP production are provided in Supplementary Fig. 5.

### Ishikawa cells display increased glycolytic reserve in hypoxia with concomitant reduction in metformin response

**Fig. 6:**
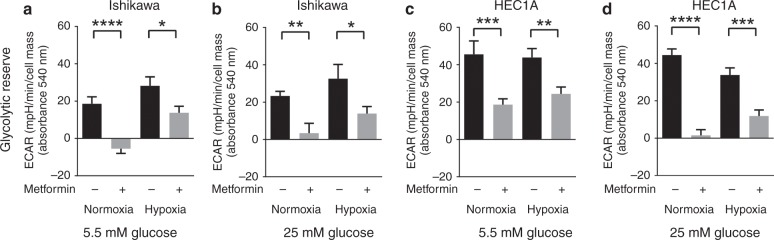
Ishikawa cells display increased glycolytic reserve in hypoxia with concomitant reduction in metformin response. Grey and black bars represented mean ± SEM with and without metformin treatment, respectively. Ishikawa cells (**a**, **b**) conditioned in low or high glucose media in hypoxia (3% O_2_) showed increased glycolytic reserve. Metformin reduced ECAR in normoxia, while more glycolytic cells showed reduced response to metformin treatment under hypoxia (3% O_2_) (**c**, **d**). A similar effect was observed in the HEC1A cell line, however cellular glycolytic reserve following metformin treatment did not differ between normoxia and hypoxia (*n* = 3 biological replicates)

The significant decrease in basal and maximal mitochondrial respiration in untreated endometrial cancer cells in hypoxia (3% O_2_) suggests a switch to glycolytic energy metabolism. Using simultaneous extracellular acidification rate (ECAR) measurements taken during the mitochondrial stress test, we were able to identify the glycolytic adaptations following oxygen, glucose and metformin-mediated mitochondrial stress. Ishikawa cells in particular, displayed increased ECAR and glycolytic reserve in hypoxia in both glucose concentrations. In both cell lines metformin has a substantial effect on reducing ECAR in normoxia (*p* ≤ 0.01–0.0001), whereas under hypoxic conditions the effect, although significant, is much less (*p* ≤ 0.05–0.001) (Fig. 6a–d). Finally, to ensure that the effects measured with the Seahorse metabolic analyser can be related directly to the toxicity results in Fig. 3, Supplementary Fig. 3, we carried out additional cell viability experiments in normoxia versus hypoxia (3% O_2_) (Supplementary Fig. 6). This confirmed that in both low and high glucose, hypoxia (3% O_2_) can attenuate the cytostatic effects of metformin (*p* < 0.05–0.0001).

## Discussion

In this study, we have shown that high glucose and hypoxia reduce metformin response both in women with endometrioid endometrial cancer and cancer cell line models. Endometrioid endometrial or Type 1 cancers represent 80% of all endometrial tumours and are thought to be driven by obesity and insulin resistance,^32^ hence the potential for metformin to act on insulin-dependent cancer signalling pathways.^7^ Metformin has shown significant promise as an anti-cancer treatment in various pre-clinical studies^25–27^ and this has been recapitulated in single arm clinical trials.^8,10,13,14^ The response to metformin, however, varied between patient groups. Indeed, we found no overall effect of metformin on Ki-67 expression in our own placebo-controlled trial (PREMIUM), however, in an *a priori* subgroup analysis, BMI modified metformin treatment effect, with non-obese women showing greater Ki-67 response.^9,33^

While metformin’s anti-cancer activity is not fully understood,^34^ its role as a mitochondrial complex I inhibitor is established.^35^ The dependence on mitochondrial respiratory activity is much reduced under hypoxic conditions, hence tumour hypoxia could influence response to metformin. High glucose concentrations are recognised to modify the effect of metformin;^28,36,37^ in breast cancer for example, mTOR inhibition by metformin was diminished in cells cultured in high glucose (11–25 mM).^38,39^ A shift towards glycolytic energy metabolism and reduced dependence on oxidative phosphorylation has been proposed as a resistance mechanism. In breast cancer cell lines, prolonged exposure to metformin reduced mitochondrial OCR with compensatory increased glycolysis.^40^

Tumour hypoxia is recognised as a poor prognostic indicator in endometrial cancer.^41^ In this study, HIF-1α and CA-9 expression on endometrial cancer biopsies were used as surrogate markers of hypoxia. Baseline HIF-1α and CA-9 were positively correlated to tumour grade, a finding that corresponds with some publications in endometrial cancer^42,43^ but not others.^44,45^ The baseline expression of HIF-1α and CA-9 was comparable in the metformin-treated and control patients. Assessing tumour hypoxia in a hysterectomy specimen can be challenging as clamping the uterine arteries and devascularising the uterus may itself contribute to increased hypoxia. The baseline hypoxia expression, however, is reliable as the live tumour is sampled and fixed in formalin immediately. Importantly, we have shown that Ki-67 response to metformin is significantly lower in tumours with higher baseline HIF-1α, suggesting decreased metformin response in hypoxic tumours.

Our in vitro studies also confirmed that endometrioid endometrial cancer cells in hypoxia and those grown in low glucose in normoxia are less responsive to the cytostatic effects of metformin. This is consistent with previous observations where metformin treatment showed a greater reduction in ovarian xenograft tumour weight in normo-glycaemic mice, compared with hyper-glycaemic mice.^46^ In breast, endometrial and ovarian cancer, low glucose conditions enhanced the cytostatic effects of metformin on cancer cells,^37,47^ while hypoxia and HIF-1α activation suppressed dichloroacetate (a glycolysis pathway inhibitor) and metformin-induced cell death.^48^ While metformin has been shown to affect apoptosis in cell culture models,^49^ our clinical studies showed that metformin treatment was not associated with an increase in apoptosis.^8,9^ Thus, we have used cytostatic models for this study.

As metformin is thought to act on mitochondrial respiration, we demonstrated that metformin treatment increases mitochondrial biogenesis, while impairing mitochondrial function. These effects were greater in low glucose using both flow cytometry and Seahorse mitochondrial stress tests. One interpretation is that high glucose (hyperglycaemia) encourages cancer cells to harness glycolytic pathways, thus protecting against a drug that targets oxidative phosphorylation.^50^ There are a number of glucose-regulated proteins in mitochondria^51^ that influence metabolism and tumour growth,^52^ suggesting that metformin treatment may cause significant alterations in mitochondrial DNA level and expression.

This is the first study to demonstrate that metformin has a direct effect on endometrioid endometrial tumour mitochondria by increasing mitochondrial mass. This increase in mitochondrial biogenesis may compensate for the effects on mitochondrial function. Further, this study is the first to report the validation and use of the Seahorse analyser under hypoxic conditions. This has allowed us to demonstrate that metformin-mediated effects on mitochondrial function are reduced in high glucose and in hypoxia, secondary to preferential glycolytic respiration. Our in vitro findings of increased mitochondrial mass were mirrored in endometrial tumour tissue from our pre-surgical study of metformin. Tumours from patients on metformin treatment had a significant increase in post-treatment TOMM20 expression, an IHC marker of mitochondrial mass. While it was not possible to directly measure mitochondrial function in the tumour samples available, it could be inferred that the increased mitochondrial mass following clinical metformin treatment is accompanied by reduced function. This mitochondrial dysfunction could contribute to the inhibition of pro-proliferative pathways and the decrease in cellular proliferation observed.

This study used translational models to explore potential mechanisms of metformin response. A strength of the pre-surgical study is the window design, which allowed the effects of metformin to be tested directly in patients, thus bypassing animal models. We were then able to confirm our findings using mechanistic cell line models. The pre-treatment endometrial biopsies, however, were scanty and finite necessitating the use of tissue micro-array. By the nature of the sampling device, the biopsies were a scrape of the tumour surface and we were thus unable to comment extensively on intra-tumoural heterogeneity of the hypoxia markers. Similarly, further studies using specifically collected endometrial cancer biopsies directly into a coating buffer are required to accurately demonstrate the effects of metformin on tumour mitochondrial function by measuring cytochrome C oxidase staining. This detects the activity of complex IV, reflecting the capacity of cells to undergo mitochondrial electron transport and oxidative phosphorylation.^53^

Hyperglycaemia and tumour hypoxia are likely to modify the cytostatic/toxic effects of metformin. A dual approach to targeting both glycolysis and oxidative phosphorylation is probably necessary to achieve cancer cell death, particularly in glucose-rich and hypoxic microenvironments. For example, the combination of short-term starvation and metformin, resulted in impaired glycolytic flux, inhibition of hexokinase II activity and impairment of oxidative phosphorylation in colon cancer cell lines and murine models.^54^ Alternatively, the addition of a glycolytic inhibitor, 2-deoxyglucose may improve metformin response.^55,56^

We set out to establish metabolic determinants of metformin response in endometrial cancer. Our data suggest that metformin may have a therapeutic role for selected patients with normoglycaemia and low grade, normoxic tumours. Hypoxic tumours were less responsive to metformin treatment, while both hypoxia and high glucose in growth media significantly reduced the cytostatic effect of metformin in vitro. An indiscriminate use of metformin in all patients with endometrial cancer is likely to result in null effects, as demonstrated by the heterogeneous response in our patients. Thus, further studies may help characterise a subset of patients most likely to derive long-term clinical benefit.

## Supplementary information

Supplementary material

## Data Availability

As data is part of a clinical trial, it is not freely available but may be requested by writing to the corresponding author.
