# Supplementary material for: Hypoxia and hyperglycaemia determine why some endometrial tumours fail to respond to metformin
Source: Br J Cancer. 2019 Dec 10;122(1):62–71. doi: 10.1038/s41416-019-0627-y (PMC6964676; doi:10.1038/s41416-019-0627-y)
Supplement: Supplementary file 1 — Supplementary material [file 41416_2019_627_MOESM1_ESM.docx]

**Supplementary Information, Table and Figure Legends**

**Supplementary Information**

Total RNA was extracted from fixed cells off the Seahorse plate following extracellular flux analysis according to the PrimeScript TMRT reagent kit (Ambion RNA) using Trizol reagent (Ambion RNA). Following DNAse treatment, total RNA (2 µg) was reverse transcribed into cDNA in a 20 μl reaction system according to the protocol of the PrimeScript TMRT reagent Kit. Real-time PCR analysis was conducted using the Applied Biosystems 7000 Sequence Detection System. The real-time PCR conditions were as follows: 50°C for 2 min followed by 95°C for 10 min, 40 cycles at 95°C for 15 s, and 60°C for 1 min. All real-time experiments were carried out in duplicate and at the end of the PCR the calculated cycle threshold (CT) values were exported to excel for analysis. Relative mRNA levels of VEGF, CA-9 and GLUT-1 from 2 different cell lines were normalized to HPRT. Primer sequences hGLUT1 Forward 5’-TGT GTA TGC CAC CAT TGG CT -3’, hGLUT1 Reverse 5’- CTA GCG CGA TGG TCA TGA GT -3, VEGF Forward 5’- ATC TTC AAG CCA TCC TGT GTG C -3’, VEGF Reverse 5’- GCT CAC CGC CTC GGC TTG T -3’, CA-9 Forward 5’- AGT TGC TGT CTC GCT TGG AA -3’, CA-9 Reverse 5’- AGA GGG CAG GAG TGC AGA TA -3’ and HPRT Forward 5’-AGG ACT GAA AGA CTT GCT CG-3’ and HPRT Reverse 5’-ATG TAA TCC AGC AGG TCA GC-3’.

**Supplementary Table 1: Primary antibodies and conditions used on Leica Bond Max**

| Primary Antibody | Manufacturer | Catalogue | Host | Additional Block | Antigen Retrieval | Dilution |
| --- | --- | --- | --- | --- | --- | --- |
| Ki-67 MIB1 | Dako | X0931 | Mouse monoclonal | 10% Casein | pH9 | 1:100 |
| HIF-1α | BD Biosciences | BD610959 | Mouse monoclonal | Not included | pH9 | 1:50 |
| CA-9 | Novus | NB100-417 | Rabbit polyclonal | Not included | pH9 | 1:2000 |
| TOMM-20 | Santa Cruz | sc-17764 | Rabbit polyclonal | Not included | pH6 | 1:250 |

**Supplementary Figures**





**Supplementary Figure 1:** Representative example of Seahorse XF Cell Mito Stress Test profile for OCR and ECAR (adapted from manufacturer’s manual)[^48^](#_ENREF_48) . (A) Mitochondrial function was assessed by sequential addition of oligomycin (OM), carbonylcyanide p-trifluoro methoxy phenyl hydrazone (FCCP) and a combination of rotenone and antimycinA (R&A). **(B)** During Mito stress test, simultaneous ECAR measurements were recorded. ECAR measurements made following OM and R&A injection represent acidification due to maximum use of their glycolytic capacity. Glycolytic Reserve = Maximum ECAR (glycolytic capacity + non-glycolytic acidification) – Basal ECAR (glycolysis + non glycolytic acidification).


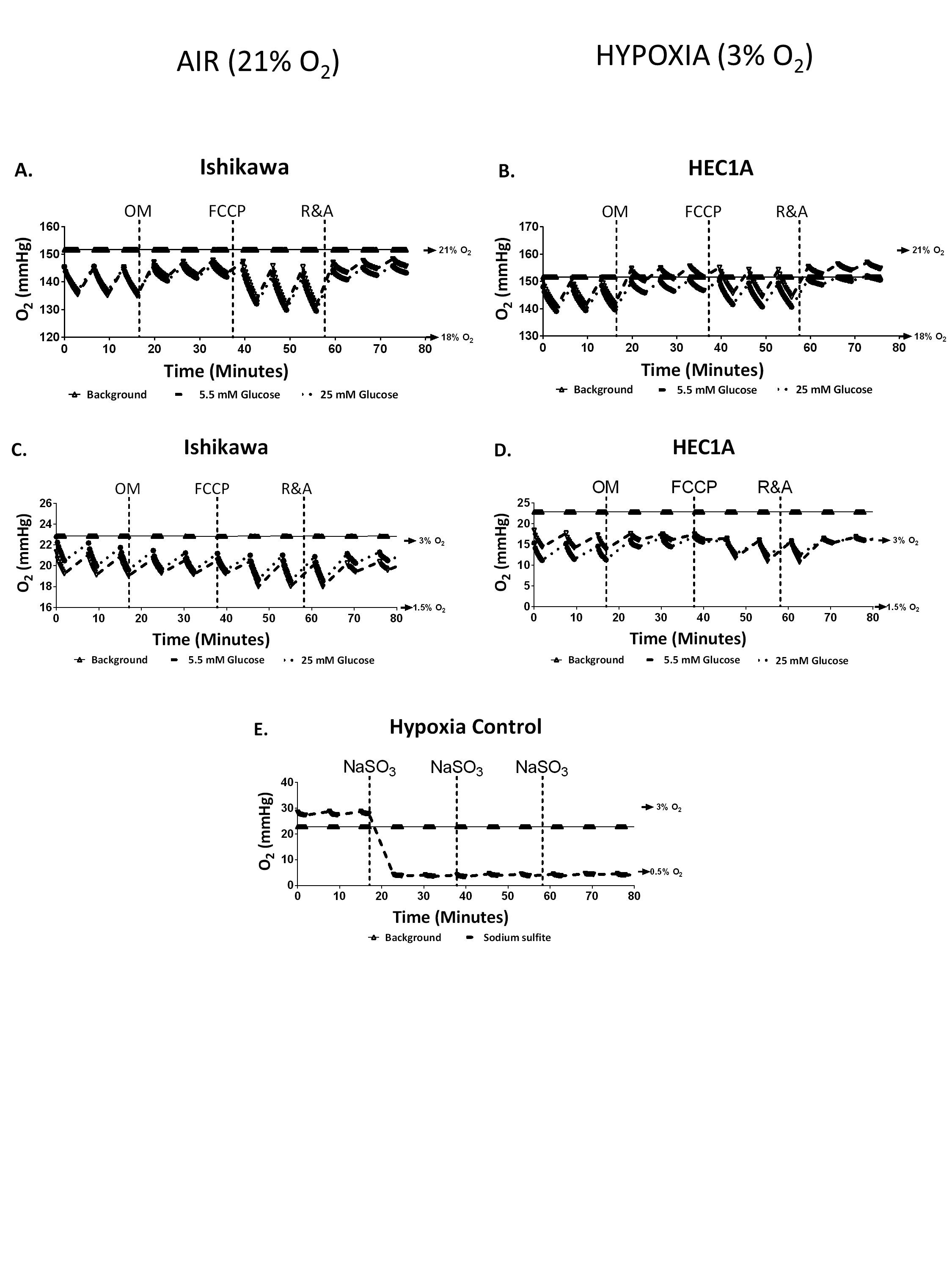


**Supplementary Figure 2:** Repeated OCR measurements require restoration of O_2_ concentrations between measurements. O_2_ levels were dependent on the available O_2_ concentrations and optimum seeding density. Figures A-D illustrate reduction in oxygen levels in response to each manipulation followed by restoration to the starting concentration during the mixing cycle. Oxygen levels in wells with Ishikawa (A&C) and HEC1A (B&D) were maintained between 150mmHg (21%) and 130 mmHg (18%) in air and between 23mmHg(~3%) and 10mmHg (1.5%) in hypoxia. In background wells (no cells), oxygen levels were constant at 150mmHg (21%) and 23mmHg (~3%), in air and hypoxia, respectively. The hypoxia control well (NaSO_3_, an oxygen scavenger) is shown in (E). This is used to confirm maintenance of hypoxia inside the i2 cabinet ; NaSo_3_ injections scavenge available oxygen and reduce levels to 5mmHg (0.5% O_2_).

**
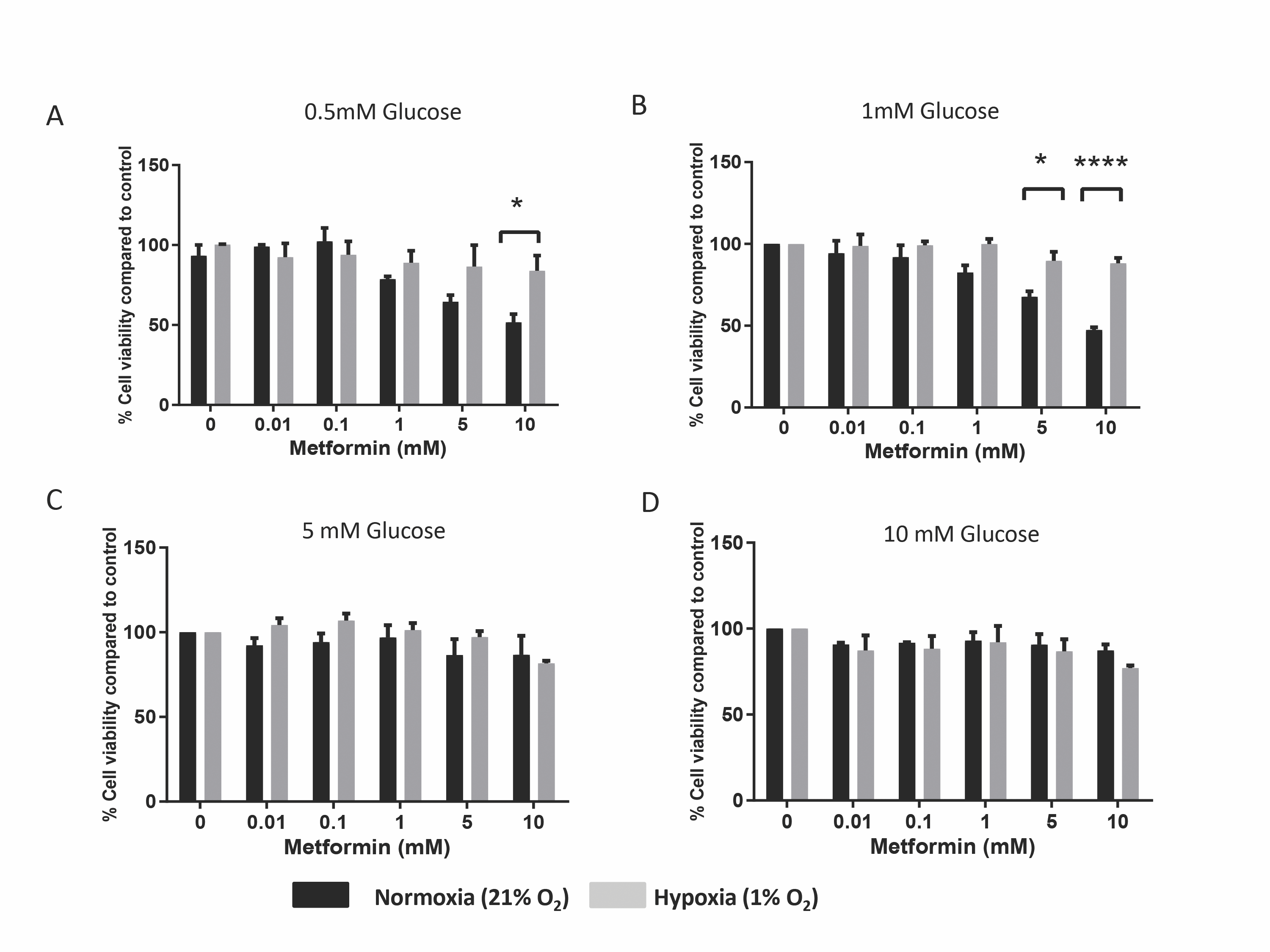
**

**Supplementary Figure 3:** Hypoxia reduces sensitivity of endometrial cancer cells to metformin grown in ultra-low glucose concentrations. Ishikawa cells in very low glucose (0.5 and 1mM) were sensitive to metformin following 24 hours of treatment. This effect was significantly reduced in hypoxia at higher concentrations of metformin (A & B). There were no differences seen in cells treated in hypoxia or normoxia. The bars represent the percentage cell viability ± SEM compared with untreated controls (n= 3 biological replicates).

**
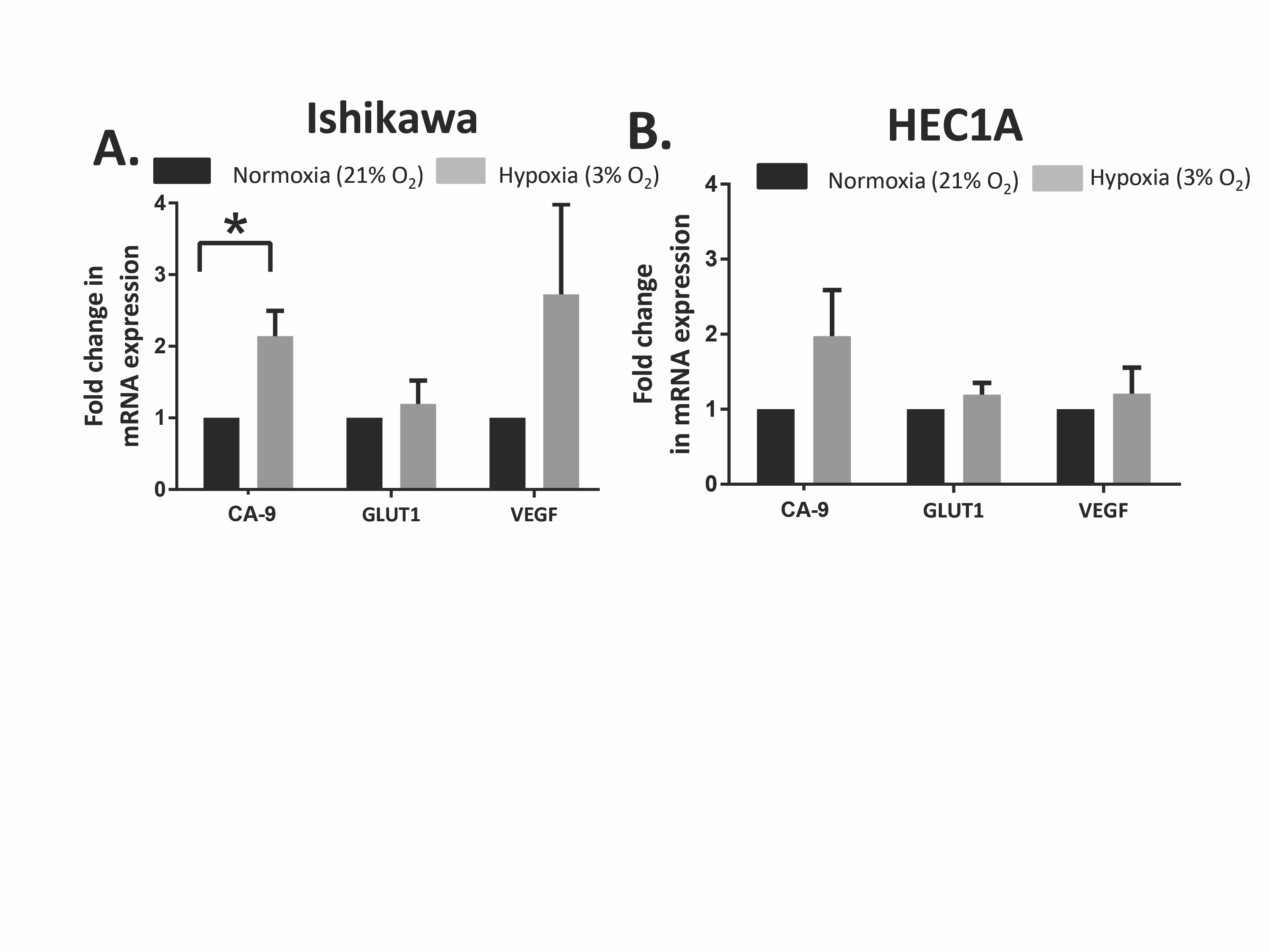
**

**Supplementary Figure 4:** RT-PCR results of RNA extracted from Ishikawa and HEC1A cell lines. Following 24 hours exposure to hypoxia (3% O2), the expression of CA-9 is increased in both Ishikawa (A) and HEC1A cell lines (B). This effect reached statistical significance in Ishikawa cell lines. GLUT1 did not show any significant increase in either of the cell lines. VEGF levels are increased in Ishikawa cell lines but this effect did not reach statistical significance. These data represent mean values ± SEM (n=3 biological replicates).


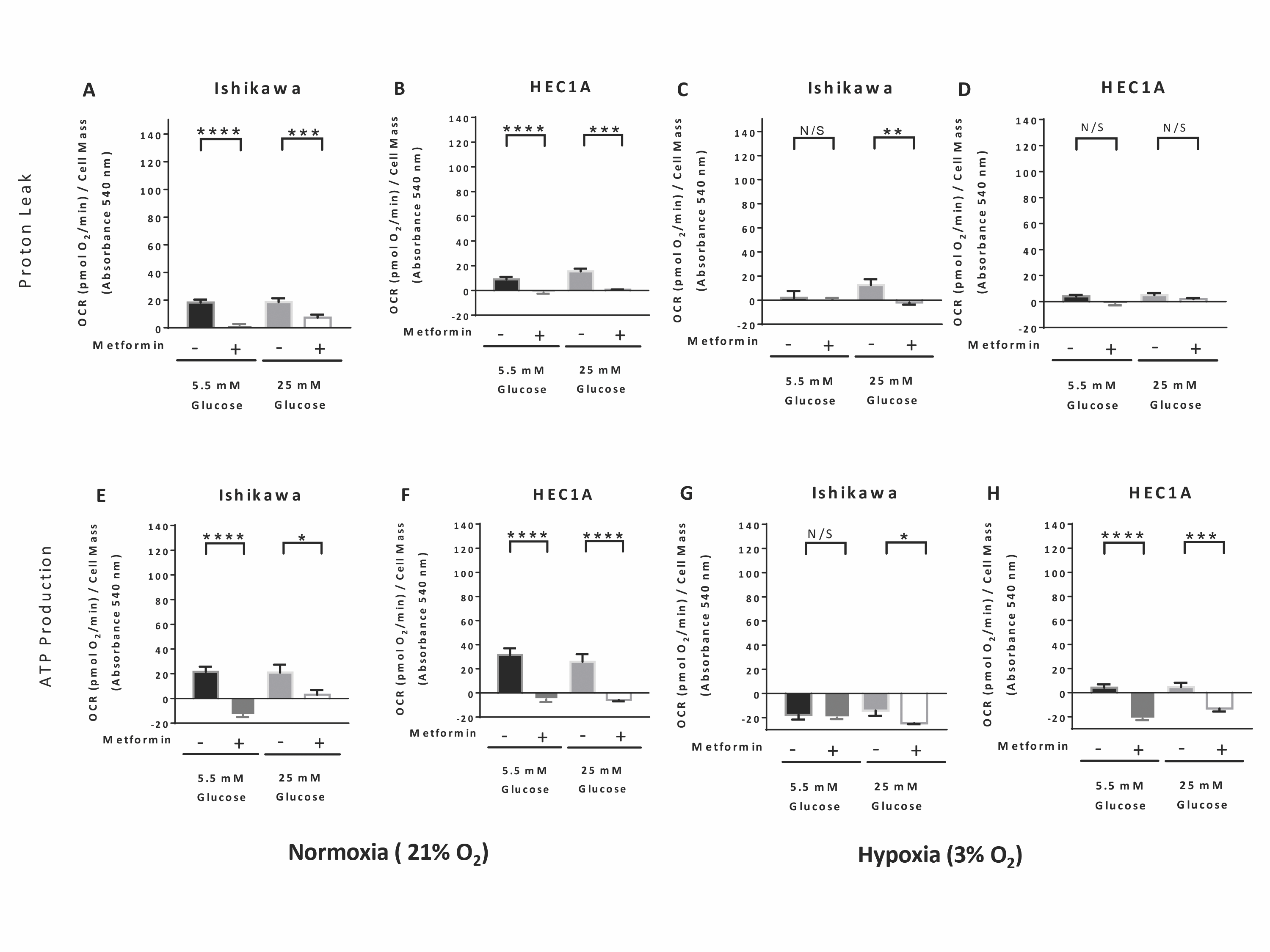


**Supplementary Figure 5:** OCR related to proton leak and ATP production is shown (A-B and E-F, respectively) in air (21% O_2_) and (C-D and G-H, respectively) in hypoxia (3% O_2_). Metformin treatment for 72 hours reduced OCR related to proton leak and ATP production. Grey and black bars represented mean ± SEM with and without metformin treatment, respectively (n=3 biological replicates).


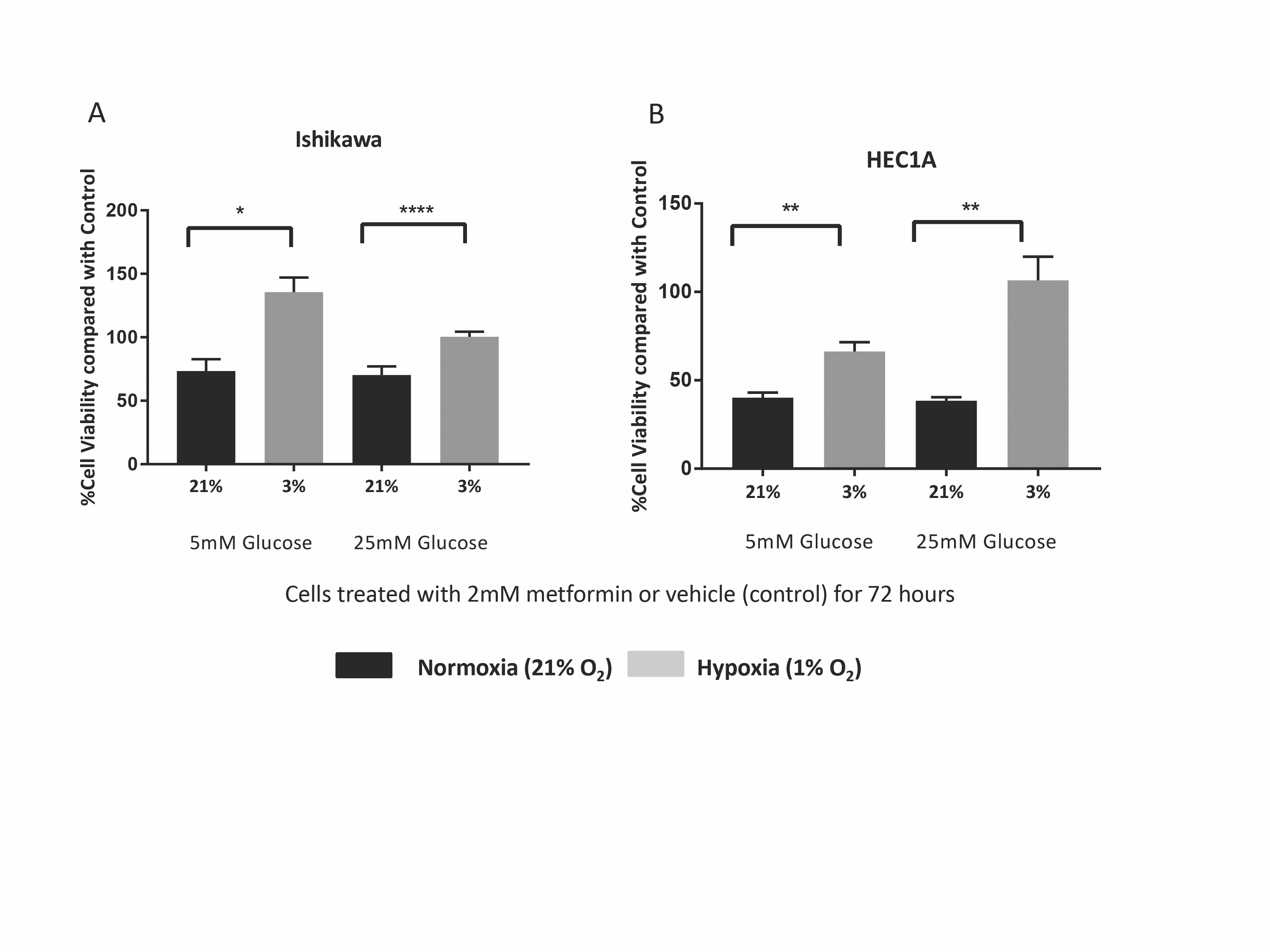
**Supplementary Figure 6:** Ishikawa and HEC1A cells were treated with metformin (2mM) or vehicle (control) for 72 hours in air or hypoxia (3%). In both low and high glucose, hypoxia attenuates the cytostatic effect of metformin (p<0.05-0.0001) (n=3 biological replicates).
